# Metabolic fingerprinting of *Lactobacillus paracasei*: the optimal quenching strategy

**DOI:** 10.1186/s12934-015-0322-5

**Published:** 2015-09-04

**Authors:** Kristina B. Jäpelt, Jan H. Christensen, Silas G. Villas-Bôas

**Affiliations:** Analytical Chemistry Group, Department of Plant and Environmental Sciences, Faculty of Science, University of Copenhagen, Copenhagen, Denmark; Chr. Hansen A/S, Hoersholm, Denmark; The Metabolomics Lab, School of Biological Sciences, University of Auckland, Auckland, New Zealand

**Keywords:** Microbial metabolomics, Propidium iodide, Glycerol saline quenching, Fast filtration, Alkylation, Silylation, Methanol quenching

## Abstract

**Background:**

Quenching in cold buffered methanol at −40 °C has long been the preferred method for sub-second inactivation of cell metabolism during metabolic fingerprinting. However, methanol is known to cause intracellular metabolite leakage of microbial cells, making the distinction between intra- and extracellular metabolites in microbial systems challenging. In this paper, we tested three quenching protocols proposed for microbial cultures: fast filtration, cold buffered methanol and cold glycerol saline.

**Results:**

Our results clearly showed that cold glycerol saline quenching resulted in the best recovery of intracellular metabolites in *Lactobacillus paracasei* subsp. *paracasei* (*L. paracasei*). Membrane integrity assayed by propidium iodide revealed that approximately 10 % of the *L. paracasei* cell membranes were damaged by contact with the cold buffered methanol solution, whilst cold glycerol saline quenching led to minimal cell damage. Due to the nature of the *L. paracasei* culture, fast filtration took several minutes, which is far from ideal for metabolites with high intracellular turnover rates.

**Conclusion:**

The implementation of a reliable, reproducible quenching method is essential within the metabolomics community. Cold glycerol saline prevented leakage of intracellular metabolites, and, thus, allowed more accurate determinations of intracellular metabolite levels.

## Background

Metabolic foot- and fingerprinting is defined as the semi-quantitative analysis of extracellular (exo-metabolome) and intracellular (the endo-metabolome) metabolites, respectively [1]. The main source of variability in the metabolic footprint is the presence of living cells in the medium, which are responsible for metabolite uptake and secretion, and secretion of extracellular enzymes. Thus, the sampling typically involves a separation of cells from medium by centrifugation or filtering. The rate of the sampling is not as critical as for fingerprinting as the turnover rate of the metabolites is slower [2]. In contrast, a prerequisite for obtaining an accurate time-preserved metabolic fingerprint is a sampling method capable of instantaneously halting (quenching) any metabolic activity in the cell [3, 4]. The effect of quenching on the metabolic fingerprint has been studied extensively [5–23]. However, the findings lack consistency as some methods are judged good by some, while inadequate by others. Slow quenching of metabolic activity, insufficient quenching efficiency, results in biases due to biochemical transformations taking place in response to the introduced environmental change during quenching. Metabolites with higher turnover rates, such as metabolites in the central metabolism, are particularly susceptible to these environmental changes. One established strategy for validating the quenching efficiency is to monitor the energy charge of the cells. The EC describes the relationship between ATP, ADP, and AMP in the cell [8, 14, 19, 24]. Alternatively, unwanted metabolic activity during quenching can be measured by mixing U^13^C labelled glucose into the quenching solution to assess changes in the labelling pattern as an indicator of remaining metabolic activity [25] or by evaluation of the consistency of the metabolic fingerprint over time [20].

A critical assumption during quenching is that the intracellular metabolites will stay inside the cells as any metabolite leakage into the extracellular space would negatively affect both accuracy (introduce a bias) and precision (a higher variability). This assumption has been increasingly questioned. Cell membrane damage has been suggested as the main reason for loss of intracellular metabolites during quenching [18, 26]. Previous studies have monitored membrane integrity during quenching by labelling of damaged cells using non-vital dyes such as propidium iodide (PI). PI intercalates with either DNA or RNA causing formation of a red fluorescent complex during loss of membrane integrity, enabling the quantification of cell damage at the single cell level [27]. An alternative measure of membrane damage is the ‘total cell damage’ which can be assessed by comparing the optical density (OD) of the treated cells to the OD value of the untreated cells [7, 16]. However, evaluation of the effects of the quenching method on membrane structure is rarely considered in metabolome studies [7, 10, 16, 21].

A frequently used method for sampling of microorganisms is fast filtration under vacuum to separate the cells from the media, followed by cell washing using a 0 °C saline solution [5, 23]. Subsequently, metabolic activity is quenched by transfer to a precooled extraction solution. The sampling time for this procedure can be high (i.e. up to several minutes) which is unsuitable for analysis of metabolites with fast turnover rates. Cold methanol quenching [60 % (v/v) at −40 °C] was proposed by de Koning and van Dam [28] for yeast cells, allowing rapid quenching of the metabolism without leakage of metabolites. Quenching in cold methanol has since become the preferred method for quenching microorganisms in metabolic fingerprinting studies [29, 30]. However, in contrast to yeast cells, intracellular metabolites have been shown to leak from bacterial cells when brought into contact with cold methanol [5, 8, 19, 23, 31]. Alternative quenching strategies that attempt to stabilise the bacterial cell during methanol quenching have been tested with mixed success. These include increasing the methanol concentration [6, 7], addition of buffers to change ionic strength [6, 8, 19, 21], and a change of culture broth:quenching solution ratios [6]. Wittmann et al. [23], Canelas et al. [6] and Taymaz-Nikerel et al. [19] established mass balances to trace the fate of metabolites with different physicochemical properties (e.g. molecular weight and polarity) by measuring metabolite levels in all sample fractions during cold methanol quenching to find the optimal quenching method. Villas-Boas and Bruheim [20] reported a method for quenching microbial cell cultures using a cold glycerol saline solution, which resulted in a more reproducible quenching with limited metabolite leakage in *P. fluorescens*, *S. coelicolor* and *S. cerevisiae*, when compared to cold methanol. Although cold glycerol saline quenching appears to be a promising quenching method, it is rarely the chosen quenching method for metabolic fingerprinting. Schaedel et al. [16] argued that the high viscosity of glycerol negatively affected the centrifugation step, and both Chen et al. [7] and Spura et al. [18] argued that part of the metabolic fingerprint was covered by a large overloaded peak when running MSTFA derivatization followed by GC–MS analysis due to residual glycerol in the extracts. Studies assessing the performance of glycerol saline quenching in respect to inactivation of metabolic activity, protection of cell structure, and prevention of leakage are limited [16, 18].

The main objective of this study was to determine the best quenching method for *Lactobacillus paracasei* subsp. *paracasei* (*L. paracasei*) cells. We compared fast filtration, cold buffered methanol, and cold glycerol saline quenching in terms of speed of quenching, the ability of the methods to protect the cell membrane judged by the PI assay, the number of identified peaks, and the precision of the peak heights obtained by methyl chloroformate (MCF) derivatization GC–MS (MCF–GC–MS). To the best of our knowledge, no previous studies have focused on quenching of *L. paracasei* and studies focusing on quenching techniques for the *Lactobacillus* genus are limited to methanol quenching by Faijes et al. [8] and Chen et al. [7].

## Results and discussion

### Fast filtration sampling of *L. paracasei*

For fast filtration, separation of cells and medium, and the saline washing, should be performed rapidly to limit changes in the metabolic fingerprint. However, the manual realization of fast filtration resulted in sampling times of several minutes due to clogging of the filter: approx. 30 s for separation of cells from medium, followed by 3–4 min of washing. As metabolism continued for several minutes under ill-defined and irreproducible conditions with respect to temperature and the availability of substrate, oxygen, and nutrients, the measured metabolic fingerprints may be biased and therefore not representative for the cultivation conditions employed. Fast filtration was discarded from further evaluation. Other studies have proposed protocols for fast filtration with sampling times less than 30–45 s from sampling to quenching [17, 23]. However, blockage of the filter is a well-known challenge [15]. Even if sampling times of 30–45 s could be obtained, fast filtration still seems unsuitable for accurate analysis of fast turnover intracellular intermediates such as pyruvate, or low-concentration metabolites such as fructose-1,6-biphosphate, and fumarate. In contrast, amino acids can be reliably sampled by fast filtration as they have longer turnover times, owing to smaller fluxes and larger pools [6].

### Impact of quenching on cell membrane integrity

**Fig. 1:**
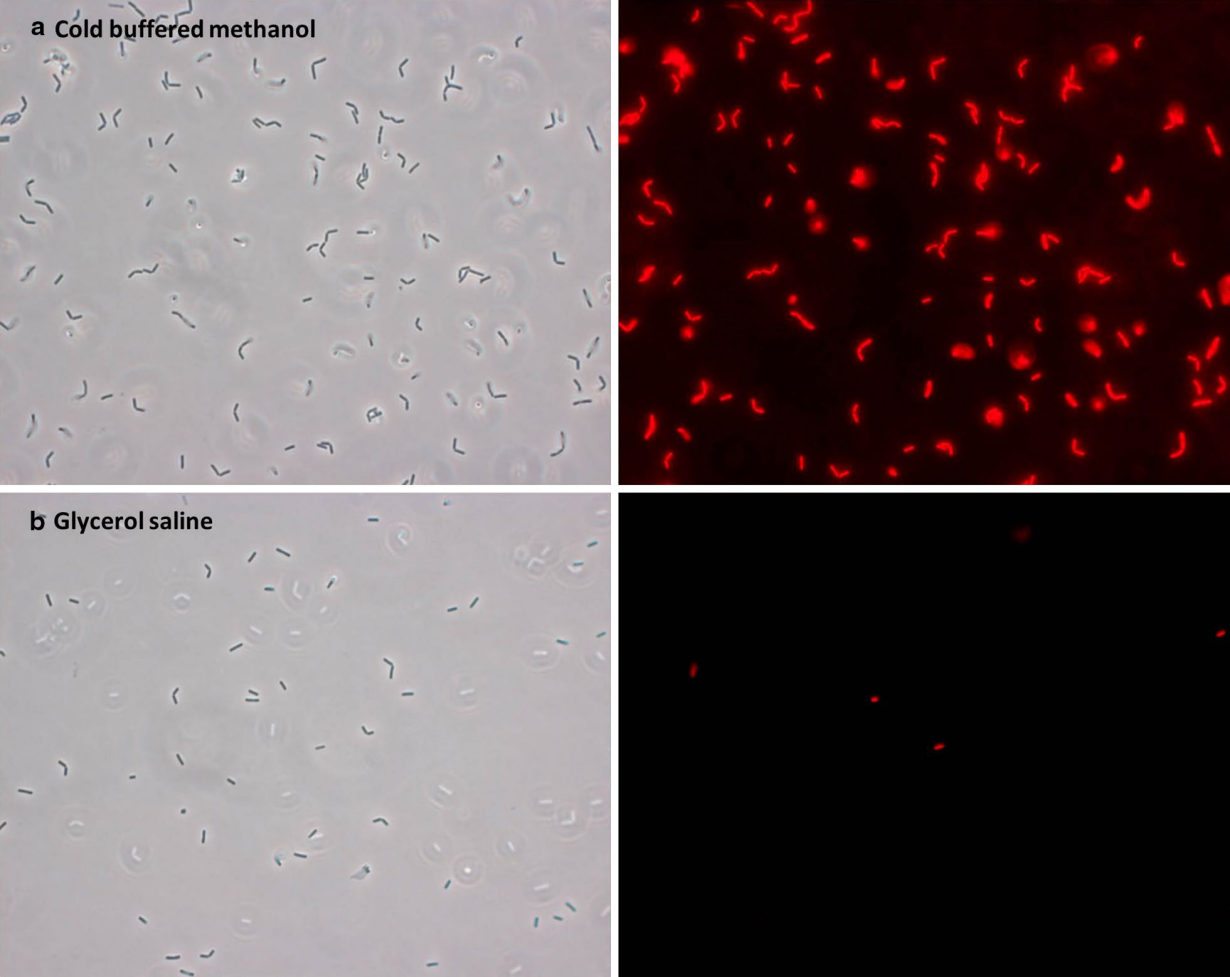
*L. paracasei* cells assayed with PI to assess cell membrane integrity during quenching. **a** Cells quenched with cold buffered methanol at −40 °C. **b** Cells quenched with glycerol saline solution at −30 °C

While it is essential to maintain an unbiased metabolic fingerprint by halting metabolic activity, a second requirement for successful quenching deals with the maintenance of cell membrane integrity. The PI membrane integrity assay showed that the *L. paracasei* cell membrane was significantly damaged by contact with buffered methanol (Fig. 1a). The damage was limited to a few cells when performing glycerol saline quenching (Fig. 1b). The variation in quenching conditions led to around 10 % PI-labelled cells for glycerol saline while nearly 100 % labelling was demonstrated for cold buffered methanol quenching.

The damaging effect of both cold buffered and unbuffered methanol quenching on the cell membrane of microorganisms has been demonstrated for *S. cerevisiae* [21], *E. coli* [16] and *L. bulgaricus* [7], whereas the protective nature of glycerol during quenching was demonstrated for *E. coli* [16] and *L. bulgaricus* [7], as limited PI labelling was observed.

### Changes in metabolic fingerprints of *L. paracasei* following quenching

**Fig. 2:**
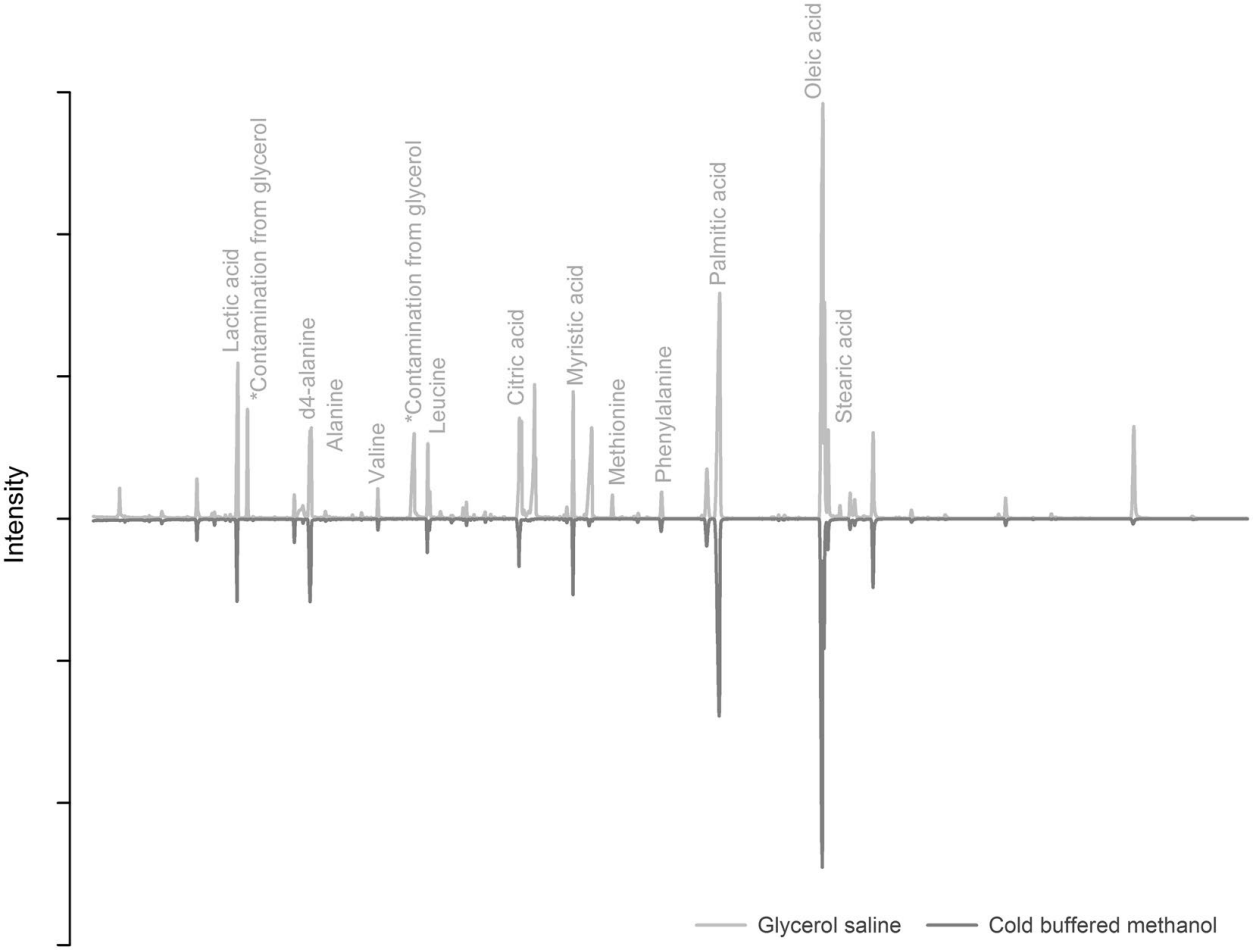
The metabolic fingerprints (TICs) of *L. paracasei* for the cold buffered methanol and glycerol saline quenching, in the retention time region from 5.6 to 35.0 min. All TICs are acquired using MCF–GC–MS, and the metabolites identified using an in-house MS library. The TIC for cold buffered methanol is inverted to ease interpretation

The loss of cell membrane integrity during cold buffered methanol quenching is expected to introduce a negative bias (i.e. underestimation of intracellular metabolites), and a lower precision of the metabolic fingerprint (i.e. increased variability). The MCF–GC–MS total ion chromatograms (TICs) after quenching with cold buffered methanol and cold glycerol saline are shown in Fig. 2. A total of 58 ± 4 and 64 ± 2 metabolites were identified for cold buffered methanol and glycerol saline (*n* = 6), respectively. The identities of some of the major peaks are marked in Fig. 2. A number of the peaks, marked with asterisks, originated from glycerol attached to the cells. Glycerol contains organic impurities, and these should be excluded from further analysis [20].

**Fig. 3:**
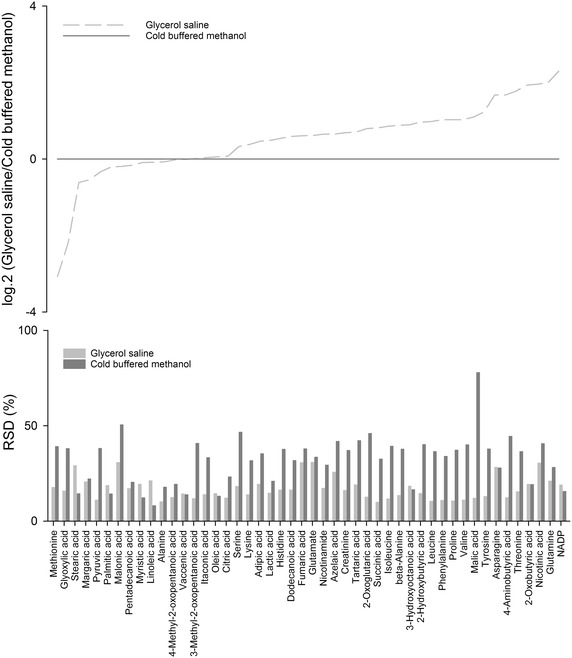
*Top* Ratio between the average biomass-normalised peak heights of glycerol saline and that of cold buffered methanol. *Bottom* RSD of the normalized peak heights for each metabolite across all biological replicates (n = 6) for cold buffered methanol and glycerol saline quenching. In this case, the abundance of each metabolite was normalized to the biomass, as well as to the total sum of peak height for all metabolites having a RSD below 20 % across all samples for both cold buffered methanol and glycerol saline quenching

A semi-quantitative comparison of the metabolite levels obtained by the two quenching methods was performed. Figure 3 (top) presents the log ratio of biomass-normalised peak heights of intracellular metabolites from glycerol saline quenching to cold buffered methanol quenching. Ratios above 0 indicates that this metabolite is present in glycerol saline quenching in a higher level than in cold buffered methanol quenching and vice versa for values below 0. In particular, the more hydrophilic compounds, such as organic acids and amino acids, were recovered in higher levels during glycerol saline quenching, whereas the longer chain fatty acids, such as palmitic acid (C16:0) and stearic acid (C18:0), were obtained in higher levels for the cold buffered methanol quenching. This might partly be attributed to the preferential extraction of the more hydrophilic metabolites by methanol through permeabilization of the membrane causing the decreased levels which is consistent with previous findings [17]. The precision of the metabolic fingerprints, as measured by the RSD of the peak heights normalized to the biomass and the total sum of reproducible peak height for all replicates, demonstrated that glycerol saline was more reproducible than cold buffered methanol. Nearly all metabolites had a higher RSD after cold buffered methanol quenching (median RSD 11 % for glycerol saline and 20 % for buffered methanol), see Fig. 3 (bottom).

### The optimal quenching method

The results show that the assumption of cell membrane integrity during cold buffered methanol quenching cannot be justified for *L. paracasei* cells (Fig. 1a). Furthermore, we observed higher biomass-normalised peak heights for glycerol saline quenching than for cold methanol quenching, which indicates leakage of intracellular metabolites and a subsequent underestimation of intracellular metabolite levels during cold methanol quenching (see Fig. 3). The protective nature of glycerol during glycerol saline quenching was demonstrated in this study, as limited PI staining was observed (Fig. 1b), biomass-normalised peak heights were higher than observed in methanol quenching, and reproducibility (as determined by RSD) was acceptable for a range of metabolite classes. The observed cell damage during cold methanol quenching can be caused by several factors with one of the more likely being changes in membrane structure induced by thermal or osmotic stress, or membrane-solvent interactions [16, 23, 32, 33]. A detailed discussion on cell damage was published by Schaedel et al. [16], and we refer to this for more information. The longer time the cells are in contact with the quenching solution, the higher the leakage will be [6, 23]; thus, the common practice during cold methanol quenching is to eliminate the washing step. However, we do not suggest this approach as the elimination of the washing step may cause significant interference of extracellular metabolites from the complex media.

Canelas et al. [6] suggested a strictly quantitative approach based on mass balances of a wide range of metabolites of different compounds classes and different physicochemical properties to trace the fate of metabolites during cold methanol treatment. Such an approach provides detailed understanding of cell leakage and metabolite loss during quenching [6, 19, 23] and may have further validated the conclusions in this study. Unfortunately, high levels of glycerol in the supernatant [approximately 50 % (v/v)] and in the washing solution, hinders mass balance calculations for the metabolites. An analysis of the metabolic levels in the supernatant and washing solution for the construction of mass balance would require an extensive cleaning step of the sample or SPE cleanup to remove the high amounts of glycerol.

### Glycerol and gas chromatography: a bad combination?

**Fig. 4:**
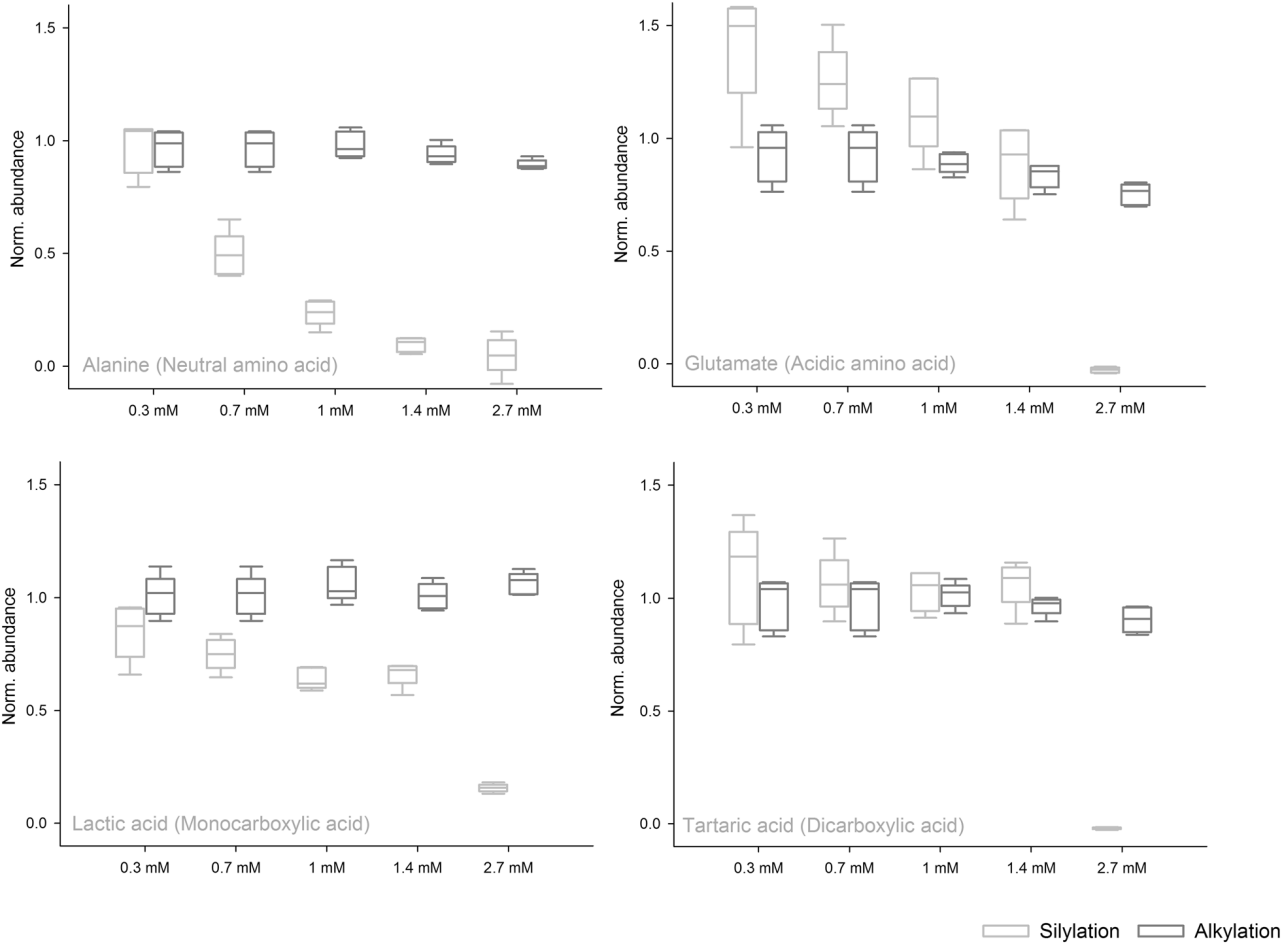
The normalized abundance of alanine, glutamic acid, lactic acid and tartaric acid as function of increasing concentrations of glycerol for both alkylation and silylation (*n* = 5). The data is normalized to the signal intensity obtained with 0 mM of glycerol. A value of 1 indicates that derivatization of the metabolite is unaffected by the increasing concentrations of glycerol

Although glycerol saline quenching seems promising for *L. paracasei*, along with other species [20], it will challenge data acquisition as glycerol cannot be fully eliminated from the final extract (~30 to 50 µL glycerol remains per sample). Non-volatile metabolites require derivatization to make them suitable for GC–MS analysis [34]. The alkylation method employed in this study converts amines and organic acids into volatile esters and carbamates, allowing them to be analysed by GC–MS. Hence, the hydroxyl groups (–OH) in glycerol does not derivatize, and the small amount of glycerol does not affect the GC–MS fingerprints. However, the classical derivatization procedure for metabolome analysis is silylation. Silylation is effective for the analysis of alcohols (including sugars and derivatives), amino acids, organic acids and fatty acids [35]. Evidently, a wider range of metabolites in the metabolome can be analysed. However, the silylation reagent react efficiently with the three hydroxyl groups in glycerol. The effect of increasing concentrations of glycerol on the metabolic fingerprint acquired by alkylation (MCF-GC-MS) and silylation (MSTFA-GC-MS) was evaluated. A highly overloaded glycerol peak was observed in the MSTFA–GC–MS chromatograms causing significant retention time shifts of metabolites (data not shown). The abundance of selected metabolites as function of the increased levels of glycerol is shown in Fig. 4.

For alkylation the abundance of all metabolites were unaffected by glycerol, while the derivatization was significantly affected by glycerol during silylation. Overall, it was clear that when all MSTFA molecules have reacted with the –OH groups in glycerol no metabolites will be detected.

## Conclusion

Significant loss of cell membrane integrity during cold buffered methanol quenching was observed for *L. paracasei* cells, along with a severe leakage of metabolites. In contrast, the use of a cold glycerol saline solution showed promising results for quenching of the cellular metabolism with the simultaneous separating of the medium components from the cell pellet, prior to metabolic fingerprinting. We demonstrated that cell membrane integrity was maintained with glycerol saline quenching, resulting in higher biomass-normalised peak heights than observed for cold buffered methanol quenching, along with a more reproducible metabolic fingerprint. However, glycerol saline quenching places restrictions on the analytical flow, as the sampling time is prolonged and the presence of glycerol leads to analytical challenges for specific data acquisition methods, especially MSTFA derivatization prior to GC–MS analysis, due to the physical (e.g. high viscosity) and chemical (e.g. hydroxyl-functional group) properties of glycerol. Developments in sample preparation and data acquisition methods are required to exploit the full potential of saline glycerol quenching for metabolic fingerprinting as combining glycerol saline quenching with MSTFA–GC–MS is of great interest.

## Methods

### Organism and inoculum

The *Lactobacillus paracasei* subsp. *paracasei* (*L. paracasei*) strain used in this study was CRL-431^®^ (Chr. Hansen A/S, Denmark). Pre-cultures were carried out by 1 % inoculation of stock solution in MRS 6.2 (Sigma-Aldrich, Germany) at 37 °C. After 16 h, pre-inoculation was repeated and the final pre-culture was inoculated at 1 % in a fermenter.

### Cultivation conditions

Anaerobic cultivations were performed in a continuous fermenter (Labfors 5, Infors HT, Switzerland). The cultivations were carried out at 37 °C with an initial pH of 6.4. Nitrogen was bubbled through the media continuously at 0.30 L min^−1^ to ensure anaerobic conditions, and the media was stirred at 100 rpm. pH and OD_600_ were continuously recorded throughout fermentation. The total feeding volume was 4 L. Feeding was initiated 10 h after inoculation at a flow rate of 0.5 mL min^−1^ and base adjustment to maintain pH at 4.4. The samples were collected when steady state was reached after a minimum of 3 volume cycles.

### Sampling for metabolic fingerprinting

The internal standard solution d_4_–alanine was added to the pellet following the addition of extraction solution. A total of 6 replicates were included for each quenching method. Pellets and extracts were stored at −80 °C until analysis was performed.

*Fast filtration* The cells were separated from the 25 mL culture medium by vacuum filtration (0.45-µm, 47 mm, Cellulose Esters, MicroScience, USA) and washed with 10 mL of 4 °C 0.9 % (w/v) NaCl solution. The biomass was rapidly (~3 to 5 s) transferred with a sterile spatula, to a 50 mL falcon tube containing 2.5 mL extraction solution maintained at −30 °C.

*Cold buffered methanol* The procedure was adapted from de Koning and van Dam [28]. A 25 mL aliquot of the cell culture broth was rapidly inactivated by transfer to the −40 °C 75 mL methanol quenching solution [60 % methanol with 0.85 % (w/v) ammonium bicarbonate (Fluka Analytical, Sigma Aldrich, Germany)]. The tube was mixed using a vortex mixer for ~5 s and placed in the pre-cooled centrifuge (10,000*g* for 10 min at −20 °C, Sorvall^®^, Thermo Scientific, USA). The supernatant was discarded; biomass pellet re-suspended and washed with 5 mL quenching solution. The centrifugation was repeated, supernatant discarded and 2.5 mL of extraction solution was added.

*Cold glycerol saline* The procedure was adapted from Villas-Boas and Bruheim [20]. A 25 mL aliquot of the cell culture broth was inactivated by mixing with 100 mL quenching solution kept at −30 °C [60 % glycerol with 0.9 (w/v) sodium chloride], and returned to the cooling bath (−30 °C, FP50-MA, Julabo, Germany), where the samples were acclimatized for 5 min. The tube was centrifuged at 36,000*g* for 20 min at −20 °C. The supernatant was discarded, biomass pellet re-suspended and washed with 5 mL washing solution (50 % glycerol saline solution). The centrifugation was repeated, the supernatant discarded, and 2.5 mL extraction solution was added.

### Extraction for metabolic fingerprinting

Each sample was thawed on ice (4 min) and mixed using a vortex mixer for 1 min, then cooled at −80 °C for 30 min. A total of three freeze–thaw cycles were performed. The sample was centrifuged at 20,800*g* for 15 min at −20 °C and the supernatant was quantitatively collected. The extraction procedure was repeated using 2.5 mL of 70 % (v/v) methanol, and the supernatants pooled. The extract was diluted prior to freeze-drying (Christ^®^ Alpha 2-4 LD plus, SciQuip, UK) by adding 20 mL of distilled water (4 °C) to enable freezing.

### Quantification of cell biomass by dry weight

Cell debris from the metabolite extraction was re-suspended in 5 mL distilled water and transferred to a pre-weighed membrane filter (0.22 µm, 47 mm, MicroScience). The tube was washed twice to remove remaining cell debris. Following vacuum filtration, the membrane filter, containing the biomass, was dried using a microwave oven (low power; 250 W for 20 min). The membrane filter was stored in a desiccator overnight, the filter weighed, and the dry weight calculated.

### PI assay

The cell pellet, after quenching, was resuspended in 1 mL 0.9 % (w/t) saline solution. 10 µL of PI (0.5 mg/mL, BioLegend, USA) was added per 0.5 mL with a cell concentration of 10^6^ cells mL^−1^. After incubation for 15 min at 4 °C, the sample was inspected using fluorescence microscopy as seen by Alfenore et al. [36]. Control samples consisted of 10 mL culture broth centrifuged and re-suspended in 0.9 % (w/v) saline solution, while the negative control consisted of culture broth microwaved for 15 s, centrifuged, and re-suspended in saline solution.

### Standard mixture for evaluation of the effect of increasing concentrations of glycerol

We selected representative compounds from various metabolic classes (Table 1). Stock solutions of standard were prepared in distilled water. Appropriate concentrations were added to obtain mixed samples which were spiked with glycerol in the concentration range from 0 to 2.7 mM. The samples were analysed using MCF-GC-MS as well as MSTFA-GC-MS. For each derivatization type 5 replicates were prepared for analysis.

**Table 1:** List of metabolite standards used to assess the effect of the analytical performance of chemical derivatization

Metabolite class	Compounds in the class
Neutral amino acids	Alanine, isoleucine, leucine, valine, glycine, asparagine, glutamine, γ-aminobutyric acid
Basic amino acids	Arginine, lysine
Acidic amino acids	Aspartate, glutamate
Aromatic amino acids	Proline, phenylalanine, tyrosine, tryptophan
Sulfur-containing amino acids	Serine, threonine, methionine
Monocarboxylic acids	Lactic acid, pyruvic acid
Dicarboxylic acids	α-Ketoglutaric acid, succinic acid, tartaric acid, malic acid, fumaric acid
Tricarboxylic acids	Citric acid
Nucleobase	Adenine, uracil, guanine, thymine, cytosine, xanthine, hypoxanthine
Nucleosides	Adenosine, 2’-deoxyadenoisine, uridine, 2’-deoxyuridine, guanosine, thymidine, cytidine, xanthosine, inosine, 2’-deoxyxanthine, adenosine triphosphate
Monosaccharides	Glucose, galactose, fructose, mannose
Disaccharides	Lactose, sucrose, maltose
Sugar alcohols	Mannitol, sorbitol
Vitamins	Thiamin, nicotinic acid, nicotinamide, pyridoxine, pyridoxamine, pantothenic acid, myo-inositol, biotin
Phosphylated sugar	Glucose-6-phosphate, fructose-1,6-biphosphate
Internal standards	d_4_-Alanine, decane, tetracosane

### Methyl chloroformate (MCF) derivatization and GC–MS analysis

The MCF derivatization method described by Smart et al. [37] was used in this study for the analysis of the metabolic fingerprintings after quenching as well as the standard mixture with increasing concentrations of glycerol. MCF derivatives were analysed using an Agilent GC7890 system interfaced to a MS 5975C inert XL MSD system (Agilent Technologies, USA). The system was equipped with a ZB-1701 GC capillary column (30 m × 250 µm i.d. × 0.15 µm with a 5 m guard column, Phenomenex). All analysis parameters were conducted according to Smart et al. [37]. 1 µL aliquots were injected in splitless mode. Injection temperature was 290 °C. Helium was used as carrier gas with a flow rate of 1 mL min^−1^. The temperature program was: 45 °C for 2 min, 9 °C min^−1^ to 180 °C and held for 5 min, followed by 40 °C min^−1^ to 220 °C and held for 11.5 min followed by a temperature ramp at 40 °C min^−1^ to 280 °C held for 2 min. The MS parameters were: filament delay of 5.5 min, mass range between 38 and 550 a.m.u., 2.85 scan s^−1^, ion source temperature 250 °C, quadrupole temperature 200 °C, and electron ionization (EI) voltage −70 eV. All samples were analysed in a randomized order.

### *N*-methyl-*N*-(trimethylsilyl)-trifluoroacetamide (MSTFA) derivatization and GC–MS analysis

20 μL standard spiked with glycerol was transferred to a GC vial with insert and the solvent evaporated under a constant flow of nitrogen at 40 °C. 20 µL of oximation reagent was added: 20 mg/mL methoxyamine hydrochloride (Supelco Analytical, USA) in 10 mL pyridine (Merch, Germany) containing 0.05 mM myristic-d_27_-acid (98 atom  % D, Isotec, USA) as surrogate standard as well as decane. Each vial was mixed and kept at 40 °C for 90 min followed by a reduction in tray temperature to 8 °C. 20 µL of *N*-methyl-*N*-(trimethylsilyl)-trifluoroacetamide (MSTFA) with 1 % chlorotrimethylsilane (TMCS) (Thermo Scientific, USA) was added and the sample incubated for 12 min at 46 °C. All sample preparation was performed using an Agilent 7693A Series Automatic Liquid Sampler. The GC (Agilent 7890A GC Agilent Technologies, USA) was equipped with a DB-5MS column (30 m × 0.25 mm, 0.25 µm film thickness; Agilent Technologies) and a 1 m retention gap (Fused silica, deactivated; 1 m × 0.25 mm; Agilent Technologies, USA). 1 µL aliquots were injected in split mode (split ratio of 5:1). The temperature was programmed as: 60 °C for 1 min, 10 °C/min to 325 °C. The BenchTOF-dx TOF MS detector (Almsco International, Germany) was controlled by ProtoTOF software (2010 Almsco International). Mass spectra were monitored between 40 and 600 *m/z* at a scan rate of 4 Hz. The ion source and transfer line temperatures were set to 230 and 290 °C, respectively, and EI was carried out at −70 eV. Data was exported to the netCDF file format.

### Data pre-processing and data analysis

Prior to data treatment, all chromatograms were inspected using MSD ChemStation E.02.02.1431. For the cellular extracts, the Automated Mass Deconvolution and Identification System (AMDIS) software was implemented for deconvoluting GC–MS chromatograms and metabolites were identified through the use of an in-house MCF mass spectral library containing over 200 spectra for the quenching samples [37]. The identifications were based on both the MS spectrum of the derivatized metabolite and its respective chromatographic retention time. The AMDIS report was used as an input to the R package IonExtractor (in-house package) along with the cdf-files to extract chromatographic peak heights for identified metabolites across all samples. The peak height for each metabolite was normalized by biomass. The ratio between average peak height for glycerol saline quenching and cold buffered methanol quenching was calculated, and further log2 transformed to adjust the variance to be the same for all intensities, see Eq. 1.

1 $$Met_{1} = { \log }2\left( { \frac{{Gly\left( {Met_{1} } \right)}}{{MeOH\left( {Met_{1} } \right)}}} \right)$$

The precision of each quenching procedure was expressed by the relative standard deviation (RSD). The peak height for each metabolite was further normalized by dividing by the total sum of peak heights for all metabolites with a RSD below 20 % across all samples for both glycerol and methanol quenching in order to remove variation not related to the relative chemical differences between quenching methods e.g. variations in injection volume, detector sensitivity and concentration.

The abundance of the metabolites in the standards running with MCF–GC–MS and MSTFA–GC–MS were determined using parallel factor analysis 2 (Parafac2) decomposing the three-way data arrays into loading matrices [38, 39]. The relative concentrations profiles, estimates of the area under each chromatographic peak, are obtained for all metabolites. The relative concentration was normalized to the height of the internal standard for both MCF–GC–MS and MSTFA–GC–MS as glycerol cannot be evaporate, thus, causing dilution effects in the sample.
