# Erratum to: Metabolic fingerprinting of *Lactobacillus paracasei*: the optimal quenching strategy

**DOI:** 10.1186/s12934-016-0413-y

**Published:** 2016-01-20

**Authors:** Kristina B. Jäpelt, Jan H. Christensen, Silas G. Villas-Bôas

**Affiliations:** Analytical Chemistry Group, Department of Plant and Environmental Sciences, Faculty of Science, University of Copenhagen, Copenhagen, Denmark; Chr. Hansen A/S, Hoersholm, Denmark; The Metabolomics Lab, School of Biological Sciences, University of Auckland, Auckland, New Zealand

## Erratum to: Microb Cell Fact (2015) 14:132 DOI 10.1186/s12934-015-0322-5

Unfortunately, the original version of this article [1] contained an error within the abstract. The sentence beginning with “Membrane integrity assayed by propidium iodide” reads “approximately 10 % of the *L. paracasei* cell membranes were damaged” but should read “approximately 100 % of the *L. paracasei* cell membranes were damaged”. The abstract has been corrected in the original article.

## References

[1] Jäpelt KB, Christensen JH, Villas-Bôas SG (2015). Metabolic fingerprinting of *Lactobacillus paracasei*: the optimal quenching strategy. Microb Cell Fact..

